# Nitric Oxide Signaling in the Auditory Pathway

**DOI:** 10.3389/fncir.2021.759342

**Published:** 2021-10-12

**Authors:** Conny Kopp-Scheinpflug, Ian D. Forsythe

**Affiliations:** ^1^Neurobiology Laboratory, Division of Neurobiology, Faculty of Biology, Ludwig Maximilian University of Munich, Munich, Germany; ^2^Auditory Neurophysiology Laboratory, Department of Neuroscience, Psychology and Behaviour, College of Life Sciences, University of Leicester, Leicester, United Kingdom

**Keywords:** auditory processing, neuronal excitability and ion channel regulation, hearing loss, neuronal nitric oxide synthase (nNOS), volume transmission, synaptic plasticity

## Abstract

Nitric oxide (NO) is of fundamental importance in regulating immune, cardiovascular, reproductive, neuromuscular, and nervous system function. It is rapidly synthesized and cannot be confined, it is highly reactive, so its lifetime is measured in seconds. These distinctive properties (contrasting with classical neurotransmitters and neuromodulators) give rise to the concept of NO as a “volume transmitter,” where it is generated from an active source, diffuses to interact with proteins and receptors within a sphere of influence or volume, but limited in distance and time by its short half-life. In the auditory system, the neuronal NO-synthetizing enzyme, nNOS, is highly expressed and tightly coupled to postsynaptic calcium influx at excitatory synapses. This provides a powerful activity-dependent control of postsynaptic intrinsic excitability via cGMP generation, protein kinase G activation and modulation of voltage-gated conductances. NO may also regulate vesicle mobility via retrograde signaling. This Mini Review focuses on the auditory system, but highlights general mechanisms by which NO mediates neuronal intrinsic plasticity and synaptic transmission. The dependence of NO generation on synaptic and sound-evoked activity has important local modulatory actions and NO serves as a “volume transmitter” in the auditory brainstem. It also has potentially destructive consequences during intense activity or on spill-over from other NO sources during pathological conditions, when aberrant signaling may interfere with the precisely timed and tonotopically organized auditory system.

## Introduction

Nitric oxide (NO) is a small molecule, highly mobile, highly reactive and soluble in water and lipid membranes, so that once synthesized it cannot be contained. While its lifetime in biological tissues may be short, its mobility permits unimpeded diffusion over significant cellular distances. The discovery of the action of “Endothelium-Derived Relaxing Factor” on vascular smooth muscle and its identification as nitric oxide earned Furchgott, Murad and Ignarro, a Nobel Prize in 1998. NO action in the brain was first linked with NMDAR-mediated increases in cGMP in the cerebellum (Garthwaite et al., 1988) and its general signaling mechanisms in the brain have been widely reviewed (Garthwaite, 2008; Friebe and Koesling, 2009; Steinert et al., 2010).

Even the NO “receptor” is unconventional, in being a cytoplasmic hemoprotein (“soluble” guanylyl cyclase, sGC) generating cGMP from GTP. Although a misnomer, we have stuck with the term “soluble” and use of “sGC” to abbreviate guanylyl cyclase. It has been shown elsewhere in the brain, including in the inferior colliculus, that the GC is actually not soluble, but anchored to PSD-95 at the synapse (Russwurm et al., 2001; Olthof et al., 2019). Indeed, the signaling cascade exhibits extreme amplification, so that physiological signaling is thought to be achieved by NO in the nanomolar concentrations (Hall and Garthwaite, 2009; Bradley and Steinert, 2015).

**FIGURE 1:**
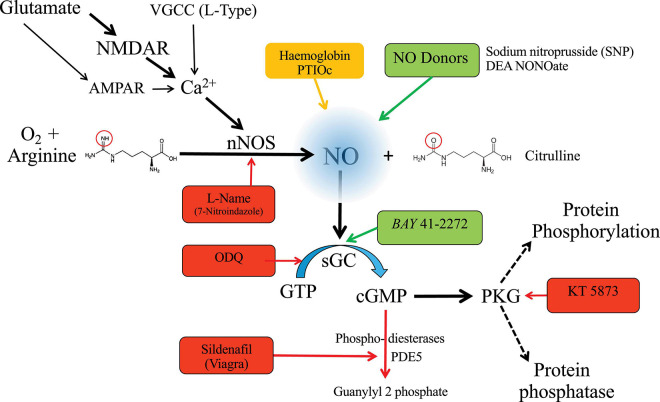
Pharmacology of nitric oxide signaling. NO is generated by glutamatergic stimulation of NMDARs, but other sources of calcium from Calcium permeable AMPAR or L-type calcium channels are also recognized. Calcium influx activates nNOS (via calmodulin) which catalyzes the conversion of the amino-acid arginine to citrulline, releasing NO. nNOS activity may be blocked by competitive antagonists such as L-NAME (NG-Nitro-L-arginine methyl ester HCl), absorbed by chelating agents, or generated independently of nNOS by perfusion of NO donors. NO diffuses across cytoplasm, membranes and between cells to bind to its intracellular receptor – soluble guanylyl cyclase (sGC) which catalyzes GTP to cGMP – a cyclic nucleotide which activates protein kinase G (PKG). ODQ (1*H*-[1,2,4]Oxadiazolo[4,3-*a*]quinoxalin-1-one) is a competitive blocker of sGC, while BAY 41-2272 is a positive modulator. KT 5873 is an antagonist of PKG. cGMP signaling may in turn be suppressed with phosphodiesterases, such as PDE5, which can be blocked by sildenafil. Blockers or antagonists are shown in red, chelating agents in orange, and positive modulators in green. The canonical pathway is indicated by the thick black arrows, with links from other sources by fine arrows, and the spectrum of PKG actions via dashed arrows.

Nitric oxide is synthetized from L-arginine and oxygen using NADPH and co-factors. This reaction is mediated by neuronal nitric oxide synthase (nNOS) in the brain. In the postsynaptic density of glutamatergic synapses, nNOS is activity-dependent and coupled through calmodulin to calcium influx at NMDARs. The canonical nNOS signaling pathway is shown in Figure 1, with examples of pharmacological agents (competitive antagonists, NO donors, sGC activators, and NO-chelating agents). The concentration of cGMP in any one cellular compartment is not only determined by the rate of production, but also by degradation through local phosphodiesterases, which further modulate signaling (Figure 1). Although cGMP may exert direct action on cyclic nucleotide-gated channels (Kaupp and Seifert, 2002) the majority of the signaling is via activation of protein kinase G (PKG) extending NO signaling capabilities, with different sGC isoforms providing important tissue-specific control (Friebe and Koesling, 2009). Facilitation of this signaling pathway is achieved by spatial proximity using cytoskeletal scaffolding proteins to bind sequential enzymes in the pathway, so nNOS is located in the postsynaptic density through PSD-95, which also binds NMDAR (Brenman et al., 1996; Christopherson et al., 1999).

Beyond the proven link to calcium influx through NMDAR, nNOS can be activated by calcium influx through calcium-permeable AMPA receptors (Haj-Dahmane et al., 2017) and L-type voltage-gated calcium channels (Pigott and Garthwaite, 2016; see Figure 1). NO signaling also modulates neuronal intrinsic excitability by acting on voltage-gated calcium, sodium, and potassium channels (Tozer et al., 2012).

Nitric oxide modulates neuronal excitability very broadly and yet nNOS knockout mice survive, as if NO is “part” of a massively redundant system (and perhaps compensated by the remaining eNOS and iNOS genes). NO signaling is highly ubiquitous in the animal kingdom (Moroz et al., 2020) and its breadth and diversity means we have yet to build consensus about its physiological roles in the nervous system. The literature has myriad observations (including those of the authors) that have yet to be consolidated into their full physiological context. The hypothesis of **retrograde NO transmission** has particularly fascinated neuroscientists, for which the evidence is reviewed elsewhere (Garthwaite, 2008). However, a presynaptic focus may have biased investigations away from other NO signaling roles: consequently, less attention has focused on **NO-mediated cGMP signaling beyond the synapse**, on kinase regulation of ion channels, and non-cGMP signaling via nitrosylation, control of gene expression or as a free radical. The auditory pathway provides a system in which many of these issues can be explored. In fact, the generation of cGMP, NO-induced intrinsic plasticity, synaptic plasticity and changes in *in vivo* firing rates have been clearly demonstrated in the auditory brainstem: cochlear nucleus: (Cao et al., 2019; Hockley et al., 2019, 2020), Superior Olivary Complex: (Steinert et al., 2008, 2011; Tozer et al., 2012; Yassin et al., 2014; Kopp-Scheinpflug et al., 2015), and Inferior Colliculus: (Olthof et al., 2019) and in an animal model of tinnitus (Coomber et al., 2014, 2015).

## Nitric Oxide Signaling Pathways in Auditory Neurons

There are **multiple elements to understanding NO signaling in the auditory system:** evidence for the presence of key signaling molecules in the pathway (nNOS/sGC/NADPH, see Table 1), identification of the target proteins and ion channels modulated, and observation of physiological/behavioral change on pharmacological intervention or genetic manipulation. This evidence must be weighed against physiological data and normal behavior since there is the potential for spill-over from other NO-generating systems and pathology, for example associated with iNOS activation during inflammatory processes. An important caveat in studying NO signaling is the extent to which an *in vitro* experimental system supports NO signaling (e.g., possessing an arginine source, NO donor validation, etc.) and whether an *in vivo* system is achieving NO activation (or inactivation) within a physiological or pathological context.

**TABLE 1:** Sites of NO signaling in the auditory pathway.

Brain area	Region/cell type	Evidence for NO-signaling	References
**Cochlea**	Inner hair cells (IHC)	Histology/physiology	Reuss and Riemann, 2000; Shen et al., 2003
	Outer hair cells (OHC)	Physiology	Shen et al., 2006
	Supporting cells	Histology	Heinrich et al., 2004
	Spiral ganglion neurons (SGN)	Histology	Fessenden et al., 1999; Vyas et al., 2019
**Cochlear nucleus**	Bushy cells of the anteroventral cochlear nucleus (AVCN)	Histology/physiology	Fessenden et al., 1999; Coomber et al., 2014, 2015; Hockley et al., 2019
	Stellate cells of the AVCN	Histology/physiology	Coomber et al., 2014; Cao et al., 2019
	Octopus cells of the posteroventral cochlear nucleus (PVCN)	Histology	Coomber et al., 2015
	Deep layers of the dorsal cochlear nucleus (DCN)	Histology/physiology	Rodrigo et al., 1994; Coomber et al., 2014
	Granule cell domain (GCD)	Histology/physiology	Coomber et al., 2015
**Superior olivary complex**	Medial nucleus of the trapezoid body (MNTB)	Histology/physiology	Rodrigo et al., 1994; Fessenden et al., 1999; Reuss and Riemann, 2000; Schaeffer et al., 2003; Steinert et al., 2008, 2011; Yassin et al., 2014; Kopp-Scheinpflug et al., 2015
	Ventral nucleus of the trapezoid body (VNTB)	Histology/physiology	Fessenden et al., 1999; Reuss and Riemann, 2000; Steinert et al., 2008
	Superior paraolivary nucleus (SPN)	Histology/physiology	Fessenden et al., 1999; Reuss and Riemann, 2000; Schaeffer et al., 2003; Steinert et al., 2008; Yassin et al., 2014; Kopp-Scheinpflug et al., 2015
	Lateral superior olive (LSO)	Histology/physiology	Rodrigo et al., 1994; Fessenden et al., 1999; Reuss and Riemann, 2000; Kopp-Scheinpflug et al., 2015
	Medial superior olive (MSO)	Histology/physiology	Reuss and Riemann, 2000; Kopp-Scheinpflug et al., 2015
**Nuclei of the lateral lemniscus**	Ventral nucleus of the lateral lemniscus (VNLL)	Histology	Rodrigo et al., 1994
	Intermediate nucleus of the lateral lemniscus (INLL)	Histology	Rodrigo et al., 1994
	Dorsal nucleus of the lateral lemniscus (DNLL)	Histology	Rodrigo et al., 1994
**Inferior colliculus**	Central nucleus of the inferior colliculus (ICc)	Histology/physiology	Olthof et al., 2019
	External cortex of the inferior colliculus (ICe)	Histology	Herbert et al., 1991; Vincent and Kimura, 1992; Coote and Rees, 2008; Keesom et al., 2018
	Dorsal cortex of the inferior colliculus (ICd)	Histology	Herbert et al., 1991; Vincent and Kimura, 1992; Coote and Rees, 2008; Keesom et al., 2018
**Medial geniculate body**	Ventral division of the medial geniculate body (MGBv)	Histology	Olucha-Bordonau et al., 2004
	Medial division of the medial geniculate body (MGBm)	Histology	Druga and Syka, 1993; Bertini and Bentivoglio, 1997
	Dorsal division of the medial geniculate body (MGBd)	Histology	Rodrigo et al., 1994; Bertini and Bentivoglio, 1997
**Auditory cortex**	Primary auditory cortex (Au1)	Histology/physiology	Wakatsuki et al., 1998; Lee et al., 2008

Adenosine 5′-triphosphate (ATP) is a major neurotransmitter and neuromodulator in the cochlea causing an increase in intracellular calcium. NO inhibits this ATP-induced calcium response via a negative feedback mechanism in inner hair cells, while at the same time enhancing the ATP-induced calcium response in outer hair cells and spiral ganglion neurons (Shen et al., 2003, 2006; Yukawa et al., 2005). Noise exposure increases nNOS expression in cochlear nucleus neurons (Coomber et al., 2014) and in spiral ganglion neurons, causing the NO concentration in the cochlea to rise from about 300 to 600 nM (Shi et al., 2002; Alvarado et al., 2016). The interaction of nNOS with activity-dependent calcium increases might be a component of the feedback in protecting inner hair cells from noise over-exposure (Shen et al., 2003; Mohrle et al., 2017). Application of nNOS inhibitors or NO donors *in vivo*, differentially affected spontaneous and sound-evoked firing rates in different cell types, which may contribute to increased gain during tinnitus (Coomber et al., 2015; Hockley et al., 2019, 2020).

There have been many studies of short-term plasticity at the giant calyx of Held synapse in the auditory brainstem (Taschenberger and von Gersdorff, 2000; Schneggenburger and Forsythe, 2006), but activity-dependent long-term plasticity has never been reported at this giant synapse. However, it is not always appreciated that NO reduces EPSC amplitudes at the calyx of Held through postsynaptic AMPAR modulation rather than a presynaptic mechanism (Steinert et al., 2008). Such a postsynaptic NO-action is corroborated by the lack of NO-modulation of presynaptic potassium currents, which would have changed transmitter release via the action potential (Wang and Kaczmarek, 1998; Yang et al., 2014). Nevertheless, other studies have demonstrated PKG-mediated modulation of synaptic vesicle endocytosis using capacitance measurements, although no change in transmitter release was reported (Eguchi et al., 2012). It is important to recognize that the probability of transmitter release, the number of release sites and rates of exocytosis and vesicle recycling are in a complex equilibrium (Hennig et al., 2008). Increased release probability (P) is “offset” by a reduced number of release sites (N) possessing fusion competent vesicles; hence after modulation the synapse may be in a different state (higher P, lower N; or lower P, higher N) even though there may be little evidence of a change in EPSC amplitude (Billups et al., 2005). Nevertheless, NO-signaling does cause an increase in spontaneous EPSCs in VCN T-stellate cells (Cao et al., 2019).

Direct effects of NO on evoked transmitter release have yet to be reported in the auditory pathway, so it is reasonable to postulate that **NO-modulation of postsynaptic neuronal excitability** (rather than synaptic mechanisms) is its primary mechanism of action. These actions may be mediated by the canonical cGMP second messenger and/or PKG-mediated phosphorylation of ion channels, for which there is direct evidence; or NO actions could be mediated by peroxynitrite formation or protein modification, such as nitrosylation (Steinert et al., 2010).

In neurons of the medial nucleus of the trapezoid body (MNTB), synaptic stimulation of the calyx of Held synapse (or perfusion of NO donors) raised cGMP and increased action potential duration, due to modulation of postsynaptic Kv3 and Kv2 potassium channels (Steinert et al., 2008, 2011). This is due to local activity-dependent generation of NO, and reciprocal modulation of potassium channel activity: so that Kv3 takes a lesser role and Kv2 takes a greater role in postsynaptic action potential repolarization, following NO signaling. This shift in intrinsic excitability reveals the hallmark of volume transmission, in that active synapses influence local quiescent neurons (having no synaptic input). This has implications for ion channel expression that follows a tonotopic gradient, such as HCN or Kv3 channels, which might be opposed (or amplified) by gradients of NO signaling, and hence ion channel activity will reflect the sum of channel expression and channel modulation (Steinert et al., 2008).

Nitric oxide also modulates HCN1 and HCN2 channels, which are differentially expressed across the superior olivary complex (Koch et al., 2004). The MNTB expresses HCN2, which has slow kinetics, while in the medial and lateral superior olive (MSO, LSO) and in the superior paraolivary nucleus (SPN), HCN channels are dominated by HCN1 subunits, which have fast kinetics. NO had distinct actions on these two channels: it facilitated HCN2 in a cGMP-dependent manner and inhibited and slowed HCN1 kinetics in a cGMP-independent manner (Kopp-Scheinpflug et al., 2015). Regulation of HCN currents is a key means of setting and regulating resting membrane potentials and the neuron membrane time-constant, since the higher Na^+^ permeability of HCN channels will drive the equilibrium to more positive potentials. In turn, a higher resting conductance generates a faster membrane time-constant, thereby modulating integration of synaptic inputs.

Another important homeostatic process is the control of intracellular chloride concentrations. A developmental shift in the chloride equilibrium potential in young animals is documented across many areas of the CNS, including the auditory brainstem. “Inhibitory” neurotransmitters such as GABA and glycine mediate depolarizing synaptic responses in neonatal animals, which become hyperpolarizing around the time of hearing onset, due to an upregulation of the potassium-chloride cotransporter 2 (KCC2; Kandler and Friauf, 1995; Lee et al., 2016). Very high levels of KCC2 (driving the chloride equilibrium to around −100 mV) are expressed in the SPN and in combination with large glycinergic inputs (from the MNTB) and high levels of HCN1 currents, enable the ionic computation of the end of a sound (Kopp-Scheinpflug et al., 2011). Activity-dependent regulation of KCC2 has been widely documented in the hippocampus and neocortex where changes in chloride gradients impact the strength of GABA_*A*_R-mediated inhibition (Chamma et al., 2013). In the SPN the strength of glycinergic inhibition is suppressed via a cGMP-dependent NO signaling at KCC2; creating a shift in the chloride equilibrium by +15 mV. This action is specific to those neurons that are expressing KCC2, which allows differential modulation of chloride reversal potentials in different neuronal populations (Yassin et al., 2014), all of which may be receiving the same inhibitory projection (for example from the MNTB).

## Discussion and Open Questions

Nitric oxide signaling is widespread, with diverse sites and convoluted actions in the nervous system. Consequently, it is often difficult to identify the source of NO signaling for a specific physiological or behavioral output, and difficult to separate physiological roles from pathological consequences, with the potential for spill-over from one synthase into the signaling system of another, e.g., iNOS to nNOS (Hopper and Garthwaite, 2006). NO is an important mediator of inflammation and pathology via up-regulation of iNOS in microglia (generating micromolar concentrations of NO). Microglia are present in the auditory brainstem, where they are involved in developmental pruning of the calyx of Held synapse (Milinkeviciute et al., 2019) and in regulating inflammation. Inflammation is associated with noise-induced hearing loss (Fuentes-Santamaria et al., 2017) and mediated by pro-inflammatory cytokines. Hearing loss and inflammation can also be caused by severe hyperbilirubinemia (Schiavon et al., 2018), where subsequent degeneration of the calyx of Held synapse is mitigated by blocking NO signaling (Haustein et al., 2010). It is worth speculating that these links between hearing loss, inflammation and NO signaling could be associated with pathological actions of microglia. The wide actions of nitric oxide, nitrosylation, nitrergic stress, and inflammation are associated with multiple neurodegenerative disease mechanisms (Bourgognon et al., 2021) and perhaps underlies broader NO mediated pathology (Steinert et al., 2010).

Nitric Oxide has a broad impact on auditory neurons and signaling. It increases evoked firing rates by enhancing intrinsic excitability, by reducing inhibitory strength and by potentiating excitatory inputs via positive feedback (Wakatsuki et al., 1998; Steinert et al., 2008; Lee, 2009; Cao et al., 2019; Hockley et al., 2019). An interesting facet of auditory signaling are high rates of spontaneous AP firing; these spontaneous rates (SRs) arise from a combination of transmitter release at inner hair cells and the intrinsic excitability of all neurons along the pathway. There is a progressive decrease in SRs from the cochlea to the cortex (Eggermont, 2015), that seems to be mirrored by higher nNOS expression in the brainstem and midbrain compared to lower nNOS expression in MGB and cortex (Druga and Syka, 1993; Olucha-Bordonau et al., 2004; Lee et al., 2008). High SRs are advantageous for temporal processing tasks in the brainstem, but are less important at higher auditory centers (such as the MGB and cortex) where auditory processing has evolved from a temporal code toward a rate code. The idea that auditory brainstem SRs carry information has been comprehensively discussed elsewhere (Litvak et al., 2003; Eggermont, 2015). While synchronization and phase-locking of AP firing are important properties of sound-evoked activity, non-sound-evoked, spontaneous firing is synchronized only during development (Babola et al., 2018) or possibly during pathological auditory signaling (Herbert et al., 1991). SRs in the healthy, mature auditory system are not synchronized. This is important because incoming sound-evoked activity defines a time window within which an action potential could be generated, intrinsic excitability permitting. So when SR is high, there is a high probability that a neuron is refractory when a sound-evoked stimulus arrives, but the stochastic distribution and desynchronization of SR between neurons maximizes the number of short latency action potentials across the population. NO-mediated modulation of SR could maintain a desynchronized SR, ensuring temporally precise and faithful transmission of responses to sound. The lower SR in higher auditory brain areas would render NO-mediated desynchronization of SR redundant, in contrast to the developing auditory system (Sonntag et al., 2009; Babola et al., 2018). An open question for the future is the extent to which activity-dependent NO signaling controls basal activity rates: a low SR before hearing onset requires little NO, and high SR on maturation needs more NO, while a stressed auditory system following noise exposure would demand even higher NO concentrations. Recruitment of NO has been shown following noise exposure (Shi et al., 2002; Zheng et al., 2006; Coomber et al., 2014, 2015; Alvarado et al., 2016) and could be involved in the development of tinnitus. The question of whether NO signaling is a cause of tinnitus or a response to correct aberrant excitability and desynchronized SR, will require future studies (Sedley, 2019).

The proposed role in desynchronizing SR might explain why NO-volume transmission does not necessarily interfere with the precise tonotopically dominated sound evoked processing. A common theme of NO action in the auditory system is the homeostatic control of excitability, be that synaptic excitation/inhibition (Wakatsuki et al., 1998; Yassin et al., 2014; Cao et al., 2019), spontaneous firing rates or neuronal intrinsic excitability. The contribution of NO to synaptic plasticity and memory formation is widely accepted in higher brain centers. Recent studies in the fruit fly have proposed that NO is more associated with active forgetting and updating of memories (Aso et al., 2019; Green and Lin, 2020). Such mechanisms might underlie auditory re-mapping following temporary hearing loss (Keating and King, 2015; Resnik and Polley, 2017). Failure to update memories in the absence of NO might also explain impaired auditory fear conditioning in nNOS knockout mice (Kelley et al., 2009).

There is strong evidence for the presence of NO signaling within the auditory brainstem. There are also broad observations of NO-mediated modulation of neuronal excitability and synaptic transmission. However, a consensus on the roles of NO in the auditory pathway has yet to be reached. Elsewhere there is ample evidence for NO involvement in synaptic plasticity, but less agreement about common downstream mechanisms. This no doubt reflects the broad signaling capabilities of cGMP and PKG (and alternate signaling by direct reactions of NO with proteins). Perhaps we need to integrate our investigations of NO signaling over a much broader range of targets (genetic, ion channel, cell signaling, metabolism/growth) in homeostasis, synaptic transmission and intrinsic excitability, and include (or control for) the potential for spill-over from pathological to physiological signaling. The superior olivary complex may lack the complexity of higher centers, but it has a well-characterized anatomy and physiology in which these complex interacting systems can be carefully explored.

## Key Concepts

• NO generation is activity-dependent and through NMDAR activation at excitatory synapses.
• Signaling involves both cGMP -dependent and -independent signaling cascades.
• NO acts by diffusion through a process of Volume Transmission to regulate excitability of neurons (including those that are active and inactive within a sphere of influence).
• NO modulates postsynaptic neuronal excitability via modulation of voltage-gated ion channels.
• Aberrant signaling underlies impaired auditory processing via changes in excitability and spontaneous firing rates.

## Author Contributions

Both authors contributed to the conception, design, and writing of the review, and have read and approved the submitted version.

## Conflict of Interest

The authors declare that the research was conducted in the absence of any commercial or financial relationships that could be construed as a potential conflict of interest.

## Publisher’s Note

All claims expressed in this article are solely those of the authors and do not necessarily represent those of their affiliated organizations, or those of the publisher, the editors and the reviewers. Any product that may be evaluated in this article, or claim that may be made by its manufacturer, is not guaranteed or endorsed by the publisher.

## Abbreviations

cGMP: cyclic guanosine monophosphate
EPSC: excitatory postsynaptic current
GABA: gamma-aminobutyric acid
GABA_*A*_R: gamma-aminobutyric acid ionotropic receptor
GTP: guanosine triphosphate
HCN: hyperpolarization-activated cyclic nucleotide gated cation channel
HCN1: hyperpolarization-activated cyclic nucleotide gated cation channel type 1
HCN2: hyperpolarization-activated cyclic nucleotide gated cation channel type 2
KCC2: potassium-chloride cotransporter type 2
LSO: lateral superior olive
MNTB: medial nucleus of the trapezoid body
MSO: medial superior olive
N: number of synaptic release sites
NADPH: nicotinamide adenine dinucleotide phosphate
NMDAR: N-methyl-D-aspartic acid or N-methyl-D-aspartate receptor
nNOS: neuronal nitric oxide synthase
NO: nitric oxide
P: release probability
PKG: protein kinase G
PSD95: postsynaptic density 95
sGC: soluble guanylate cyclase
SPN: superior paraolivary nucleus
SR: spontaneous rate.
